# Prognostic Value of Contrast-enhanced Cardiac Magnetic Resonance Imaging in Patients with Newly Diagnosed Non-Ischemic Cardiomyopathy: Cohort Study

**DOI:** 10.1371/journal.pone.0057077

**Published:** 2013-02-20

**Authors:** Karin A. L. Müller, Iris Müller, Ulrich Kramer, Reinhard Kandolf, Meinrad Gawaz, Axel Bauer, Christine S. Zuern

**Affiliations:** 1 Medizinische Klinik III, Kardiologie und Kreislauferkrankungen, Eberhard Karls University, Tübingen, Germany; 2 Radiologische Universitätsklinik, Eberhard Karls University, Tübingen, Germany; 3 Molekulare Pathologie, Eberhard Karls University, Tübingen, Germany; The University of Texas Health Science Center, United States of America

## Abstract

**Background:**

Owing to its variable course from asymptomatic cases to sudden death risk stratification is of paramount importance in newly diagnosed non-ischemic cardiomyopathy. We tested whether late gadolinium enhancement (LGE) assessed by cardiac magnetic resonance (CMR) imaging is a prognostic marker in consecutive patients with newly diagnosed non-ischemic cardiomyopathy.

**Methods:**

We enrolled 185 patients who presented for evaluation of newly diagnosed non-ischemic cardiomyopathy. Coronary artery disease was excluded by coronary angiography. Following risk markers were additionally assessed: NYHA functional class (≥II), brain natriuretic peptide (>100 ng/l), troponin I (TnI, ≥0.03 µg/l), left ventricular ejection fraction (LVEF, ≤40%), left ventricular enddiastolic diameter (>55 mm) and QRS duration (>98 ms). Endpoint of the study was the composite of all-cause mortality, heart transplantation, aborted sudden death, sustained ventricular tachycardia or hospitalization due to decompensated heart failure within three years of follow-up.

**Results:**

During median follow-up of 21 months, 54 patients (29.2%) reached the composite endpoint. Ninety-four of the 185 patients (50.8%) were judged LGE-positive. Prognosis of LGE-positive patients was significantly worse than that of LGE-negative patients (cumulative 3-year event rates of 67.4% in LGE-positive and 27.2% in LGE-negative patients, respectively; p = 0.021). However, in multivariable analysis, presence of LGE was not an independent predictor of outcome. Only LVEF ≤40% and TnI ≥0.03 µg/l were independent risk predictors of the composite endpoint yielding relative risks of 3.9 (95% CI 1.9–8.1; p<0.0001) and 2.2 (95% CI 1.2–4.0; p = 0.014), respectively.

**Conclusions:**

In consecutive patients presenting with newly diagnosed non-ischemic cardiomyopathy, LGE-positive patients had worse prognosis. However, only traditional risk parameters like left ventricular performance and cardiac biomarkers but not presence of LGE were independent risk predictors.

## Introduction

Newly diagnosed non-ischemic cardiomyopathy is a frequent diagnosis in patients with acute heart failure symptoms or reduced systolic left ventricular function. Clinical manifestations of patients with newly diagnosed non-ischemic cardiomyopathy vary, with a wide spectrum of symptoms ranging from asymptomatic courses or chest pain to severe illness with cardiogenic shock [1]–[3]. In contrast to ischemic heart failure, the underlying cause of myocardial disease and its prognostic outcome often remain unclear. The patient may recover, develop stable chronic heart failure or will require further invasive therapy including cardiac resynchronization [4], [5], implantation of assist devices [6] or heart transplantation [7]. Therefore, risk stratification of the individual patient presenting with newly diagnosed non-ischemic cardiomyopathy is of crucial importance to control the clinical course of the disease.

The predictive value of late gadolinium-enhanced (LGE) cardiac magnetic resonance imaging (CMR) has been shown in different entities of chronic myocardial disease like ischemic heart disease [8], [9], dilated cardiomyopathy (DCM) [10], hypertrophic cardiomyopathy (HCM) [11] and viral myocarditis [12]. However, little is known about the prognostic power of LGE for prediction of outcome in a real-world clinical setting of consecutive patients presenting with newly diagnosed non-ischemic cardiomyopathy.

Aim of the present study was to investigate the prognostic value of LGE together with clinical, biochemical and left ventricular risk markers in unselected patients with newly diagnosed non-ischemic cardiomyopathy.

## Methods

### Recruitment and Follow-up

Patients were enrolled if they presented with newly diagnosed (<4 weeks) non-ischemic heart failure symptoms and recent findings suggestive of cardiac structural damage (impaired global or regional left ventricular function, left ventricular enlargement, increase of cardiac enzymes, pericardial effusion or electrocardiographic (ECG) abnormalities such as non-sustained or sustained ventricular tachycardia). Significant coronary artery disease (CAD) (>50% diameter luminal stenosis of two or more epicardial vessels or left main or proximal left anterior descending coronary artery stenosis >50% [13]) was ruled out by coronary angiography in all patients before enrollment into the study. Patients with history of myocardial infarction or ischemic scar on CMR as a sign of unrecognized myocardial damage due to CAD were excluded. Indication for further diagnostic work-up such as endomyocardial biopsy for suspected myocarditis or storage disease was based on the individual decision of the treating cardiologist. All patients received medication according to current ESC and ACC/AHA guidelines depending on their left ventricular function and heart failure symptoms [14]. The study was approved by the local ethics committee of the University Hospital of Tuebingen (project number 95/2009BO1) and patients gave written informed consent.

Minimum follow-up was 6 months with clinical appointments every 6 months in our outpatient clinics where physicians were blinded to the patients` study participance. Patients who failed to meet these appointments were contacted by telephone or letter at the corresponding intervals. None of the patients was lost to follow-up.

### Endpoints of the Study

The endpoint of this study was the composite of all-cause mortality, heart transplantation, aborted sudden death (successful cardiopulmonary resuscitation or appropriate discharge of implanted cardioverter-defibrillator (ICD)), sustained ventricular tachycardia (defined as ventricular extrasystoles >120 beats per minute for >30 seconds documented in implanted devices or in Holter recordings) or hospitalization due to decompensated heart failure. In case of a suspected event, all necessary medical records were reviewed by an independent endpoint committee.

### Assessment of Left Ventricular Risk Markers by Contrast-enhanced CMR

CMR was performed on a 1.5 Tesla (T) scanner (Siemens Medical Systems, Germany) providing a gradient strength of 40 mT/m and maximum slew rate of 200 mT/m/msec. An advanced cardiac software package was used. Images were acquired with the subject in the supine position, by applying electrocardiographically gated breath-hold sequences.

To evaluate functional parameters, the protocol included a breath-hold steady-statefree-precession (SSFP) pulse sequence (repetition time/echo time 3.0/1.5 ms; flip angle 60°, 25 frames per cardiac cycle, matrix 256×192, field of view 300–400 mm) used to acquire cine images in 2-chamber, 4-chamber, short-axis, as well as outflow tract orientation of the right and left ventricle. A stack of contiguous short-axis slices from ventricular apex to base (slice thickness 5 mm, gap 5 mm) was obtained, parallel to the atrioventricular groove, covering the entire left and right ventricle.

Quantitative analysis of functional parameters was performed off-line using dedicated software (ARGUS, Siemens Medical Systems, Germany). End-diastolic volumes (EDV) and end-systolic volumes (ESV) were used to determine left ventricular ejection fraction (LVEF: EDV-ESV/EDV×100). Left ventricular short axis diameter was measured on a midventricular slice position. Left ventricular enddiastolic diameter (LVEDD) was measured using the short-axis slice at the level of the tip of the mitral valve leaflets.

For LGE imaging a two-dimensional inversion-recovery segmented k-space gradient-echo MR sequence was performed with the following parameters: repetition time/echo time/inversion time 8.0/4.9/240.0–300.0 ms, flip angle 30°, section thickness 8 mm, in-plane resolution 1.2×1.5 mm. For all examinations, the optimal inversion time to suppress the signal of normal myocardium was determined with an inversion recovery prepared SSFP sequence with incrementally increasing inversion times (repetition time/echo time 24/1.12 ms, flip angle 60°, section thickness 8 mm, and inversion times increasing in 20.0 ms increments). CMR images were acquired in short- and long-axis views 10–15 minutes after intravenous injection of 0.15 mmol per kilogram of body weight gadobutrol (Gadovist, Bayer Healthcare, Germany). Total examination time was between 30–45 minutes.

Two experienced investigators independently reviewed the image loops of each subject in a random fashion. For LGE image analysis both readers visually judged the occurrence (presence versus absence), localization, and pattern of LGE. Pattern and extent of LGE were assessed by using short- and long-axis views and were defined as present only if they were detectable in two orthogonal planes. Areas of LGE were allocated to the American Heart Association 17-segment model.

### Assessment of Additional Risk Markers

Cardiac biomarkers included brain natriuretic peptide (BNP) and troponin I (TnI) which were assessed by immunoassay (ADVIA Centaur BNP Assay and ADVIA Centaur TnI-Ultra). QRS duration was assessed from standard 12-lead ECG at study entry which were recorded at a paper speed of 50 mm/s.

### Statistical Analysis

Continuous variables are expressed as mean ± standard deviation and compared using Mann–Whitney U- test. Categorical data are presented as proportions and analyzed by chi-square test. Cox proportional-hazards regression analysis was performed to assess the association of clinical, biochemical and left ventricular risk markers with endpoint occurrence. For this analysis, continuous variables were dichotomized as follows: NYHA functional class ≥II, BNP>100 ng/l, TnI≥0.03 µg/l, LVEF≤40%, LVEDD>55 mm and QRS>the median of 98 ms. After univariable analysis, statistically significant variables (p<0.05) were forced to enter the multivariable model which was adjusted for age and gender. Survival curves of patients were calculated by Kaplan-Meier analyses and compared with Log-Rank test. Time point for begin of survival analysis was the date of CMR. Comparisons were considered statistically significant if two-sided P value was <0.05. Statistical analyses were performed using SPSS software version 19.0 (SPSS Inc., Chicago, IL, USA).

## Results

### Clinical Characteristics

185 consecutive patients presenting with newly diagnosed non-ischemic cardiomyopathy were enrolled. After complete diagnostic work-up, 102 (55.1%) were suspected to have DCM, 65 patients (35.1%) to suffer from acute, subacute or chronic myocarditis, 15 patients (8.1%) to have hypertrophic obstructive cardiomyopathy (HOCM), hypertrophic non-obstructive cardiomyopathy (HNCM) or hypertensive cardiomyopathy and three patients (1.6%) to have storage disease (table 1).

**Table 1:** Suspected etiologies of newly diagnosed non-ischemic cardiomyopathy in our study population.

All patients	n = 185
Dilated cardiomyopathy	102 (55.1)
(Sub) acute or chronic myocarditis	65 (35.1)
HNCM/HOCM or hypertensive heart disease	15 (8.1)
Storage disease	3 (1.6)

Values are n (%).

HNCM – hypertrophic non-obstructive cardiomyopathy, HOCM – hypertrophic obstructive cardiomyopathy.

Patients` baseline characteristics are presented in table 2. Mean age of the study population was 51.2±15.9 years. One quarter of patients (28.6%) were female.62.2% of the patients presented with moderately or severe symptoms of heart failure (NYHA functional class ≥II). Patients presented with a mean BNP of 733.1±1360 ng/l (normal range<100 ng/l), C-reactive protein of 1.7±3.6 mg/dl (normal range <0.5 mg/dl), CK of 258±635U/l (normal range <170U/l) and TnI of 0.4±1.7 µg/l (normal range<0.1 µg/l). Mean LVEF was 43.3±16.0%. Mean LVEDD was 51.1±9.7 mm. Mean QRS duration was 103±23 ms.

**Table 2:** Patients` baseline characteristics.

Parameters	Value (n = 185)
*Clinical risk markers*	
Mean age, y ± SD	51.2±15.9
Gender, female	53 (28.6)
NYHA-class ≥II	115 (62.2)
*Biochemical risk markers*	
BNP (ng/l)	733.1±1360
CRP (mg/dl)	1.7±3.6
CK (U/l)	258.0±635
TnI (µg/l)	0.4±1.7
*Left ventricular risk markers*	
LVEF (%)	43.3±16.0
LVEDD (mm)	51.1±9.7
Presence of LGE	94 (50.8)
Localization of LGE	
anterior wall	13 (7.0)
posterior wall	16 (8.6)
lateral wall	12 (6.5)
Septal	38 (20.5)
multifocal left ventricular	9 (4.9)
right ventricle	6 (3.2)
Pattern of LGE	
midwall LGE	83 (44.9)
Non-midwall LGE	11 (5.9)
*Electrocardiographic risk marker*	
QRS duration (ms)	103±23
*Cardiac Medication*	
ß-Blockers	165 (89.2)
ACE-Inhibitors	150 (81.1)
ATI-Antagonists	25 (13.5)
Diuretics	145 (78.4)
Aldosteroneantagonists	110 (71.0)

Values are n (%) or mean±standard deviation. BNP – brain natriuretic peptide, CMR – cardiac magnetic resonance imaging, CRP – C-reactive protein, CK – creatinkinase, DCM –dilated cardiomyopathy, HCM – hypertrophic cardiomyopathy, LGE – late gadolinium enhancement, LVEDD – left ventricular enddiastolic diameter, LVEF - left ventricular ejection fraction, NYHA – New York Heart Association, SD – standard deviation, TnI –troponin I, y – years.

**Figure 1:**
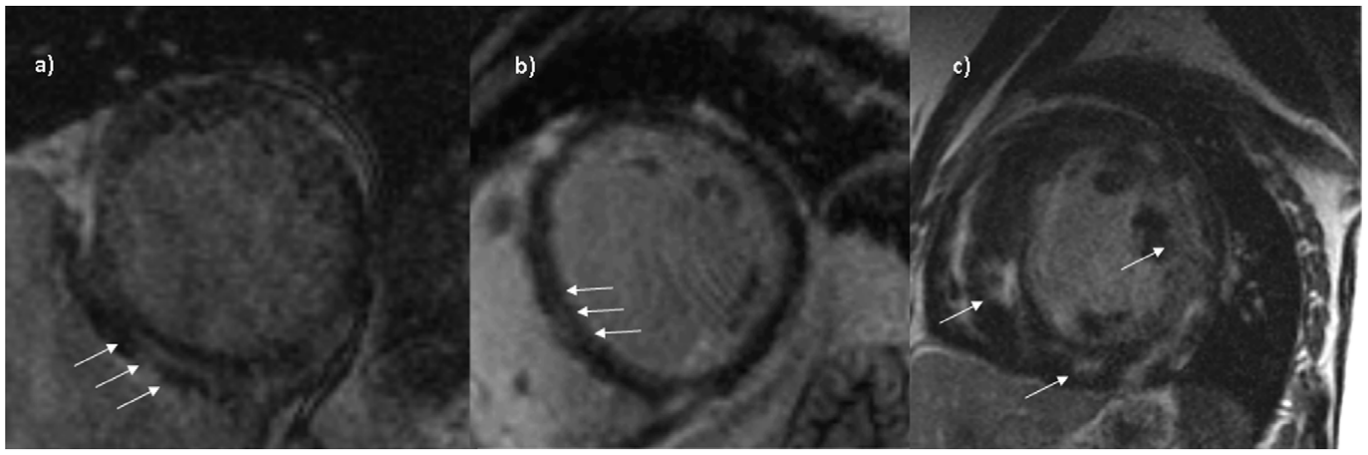
Late gadolinium-enhanced (LGE) cardiac magnetic resonance imaging in short-axis orientation of the left ventricle in three different patients. Different types of LGE can be found, characteristic for entity of non-ischemic cardiomyopathy. (a) Epicardial LGE in a patient suspected of having (sub)acute myocarditis. (b) Non-ischemic cardiomyopathy with diffuse midwall stripe pattern of the septum, indicating idiopathic dilated cardiomyopathy. (c) Typical patchy LGE/fibrosis of the septal as well as the free lateral wall segments seen in hypertrophic cardiomyopathy.

LGE was present in 94 of the 185 patients (50.8%). Among those, LGE was most commonly located in the interventricular septum (n = 38, 20.5%). LGE was detected in the left ventricular anterior wall in 13 patients (7.0%), in the posterior wall in 16 patients (8.6%), in the lateral wall in 12 patients (6.5%), in the right ventricle in 6 patients (3.2%). Nine patients (4.9%) exhibited multifocal left ventricular LGE. 83 patients (44.9%) exhibited a mid-wall enhancement while 11 (5.9%) showed a non-midwall enhancement pattern. Figure 1 displays typical CMR results.

89.2% and 81.1% of patients were treated with ß-Blockers and ACE-Inhibitors respectively, 13.5% were on AT1-antagonists, 78.4% received diuretics and 71.0% were taking aldosterone antagonists. Within the time of follow-up ICD implantation was performed in 57 patients (30.8%). Of those, 19 patients received cardiac resynchronization therapy (10.3%).

At baseline, LGE-positive patients were characterized by a significantly lower LVEF as compared to patients without LGE (39.2±15.8% and 47.5±15.2%, p<0.0001, respectively). LVEDD was significantly higher in LGE-positive patients (52.5±9.8 mm vs. 49.6±9.4 mm, p = 0.039). Furthermore, LGE-positive patients had higher serum levels of BNP (896±1499 ng/l vs. 532±1146 ng/l, p = 0.014). No differences between LGE-positive and negative patients were observed for age, gender, QRS duration and cardiac medication (table 3).

**Table 3:** Baseline characteristics of patients with and without late gadolinium enhancement.

Parameters	Patients with LGE (n = 94)	Patients without LGE (n = 91)	*P*
*Clinical risk markers*			
Mean age, y ± SD	51.5±18.0	50.8±13.4	0.605
Gender, female	22 (23.4)	31 (34.1)	0.143
NYHA-class ≥II	63 (67.0)	52 (57.1)	0.176
*Biochemical risk markers*			
BNP (ng/l)	896±1499	532±1146	0.014
CRP (mg/dl)	1.8±4.2	1.6±2.7	0.217
CK (U/l)	226±384	292±823	0.671
TnI (µg/l)	0.6±2.2	0.3±0.8	0.108
*Left ventricular risk markers*			
LVEF(%)	39.2±15.8	47.5±15.2	<0.0001
LVEDD (mm)	52.5±9.8	49.6±9.4	0.039
*Electrocardiographic risk marker*			
QRS duration (ms)	102±22	104±24	0.688
*Cardiac Medication*			
ß-Blockers	85 (90.4)	80 (87.9)	0.251
ACE-Inhibitors	76 (80.9)	74 (81.3)	0.590
ATI-Antagonists	14 (14.9)	11 (12.1)	0.454
Diuretics	70 (74.5)	75 (82.4)	0.319
Aldosteroneantagonists	56 (59.6)	54 (59.3)	0.569

Values are n (%) or mean±standard deviation. BNP – brain natriuretic peptide, CMR – cardiac magnetic resonance imaging, CRP – C-reactive protein, CK – creatinkinase, LGE – late gadolinium enhancement, LVEDD – left ventricular enddiastolic diameter, LVEF - left ventricular ejection fraction, NYHA – New York Heart Association, SD – standard deviation, TnI – troponin I, y – years.

### Follow-up and Outcome

During median follow-up period of 21 months, 54 patients of the 185 patients (29.2%) reached the composite endpoint (table 4). 10 patients (5.4%) died, all for cardiac reasons. Three patients (1.6%) underwent heart transplantation for end-stage progressive heart failure. Two patients (1.1%) were successfully resuscitated due to cardiac arrest. Adequate discharge of ICD occurred in 18 patients (9.7%). Sustained ventricular tachycardia was documented in 24 (13.0%) patients. 17 patients (9.2%) were admitted for treatment of decompensated heart failure.

**Table 4:** Clinical outcome during follow-up.

Parameters	All patients	Patients with LGE (n = 94)	Patients without LGE (n = 91)	*P*
Composite endpoint*	54 (29.2)	35 (37.2)	19 (20.9)	0.014
All-cause death	10 (5.4)	6 (6.4)	4 (4.4)	0.550
Heart transplantation	3 (1.6)	2 (2.1)	1 (1.1)	0.580
Aborted sudden death	20 (10.8)	16 (17.0)	4 (4.4)	0.006
Sustained VT	24 (13.0)	18 (19.1)	6 (6.6)	0.011
HF- related rehospitalization	17 (9.2)	8 (8.5)	9 (9.9)	0.745

Values are n (%).

* all-cause death, heart transplantation, aborted sudden death, sustained ventricular tachycardia, hospitalization due to decompensated heart failure.

HF – heart failure, LGE – late gadolinium enhancement, VT – ventricular tachycardia.

### Association of Risk Predictors with Outcome

**Figure 2:**
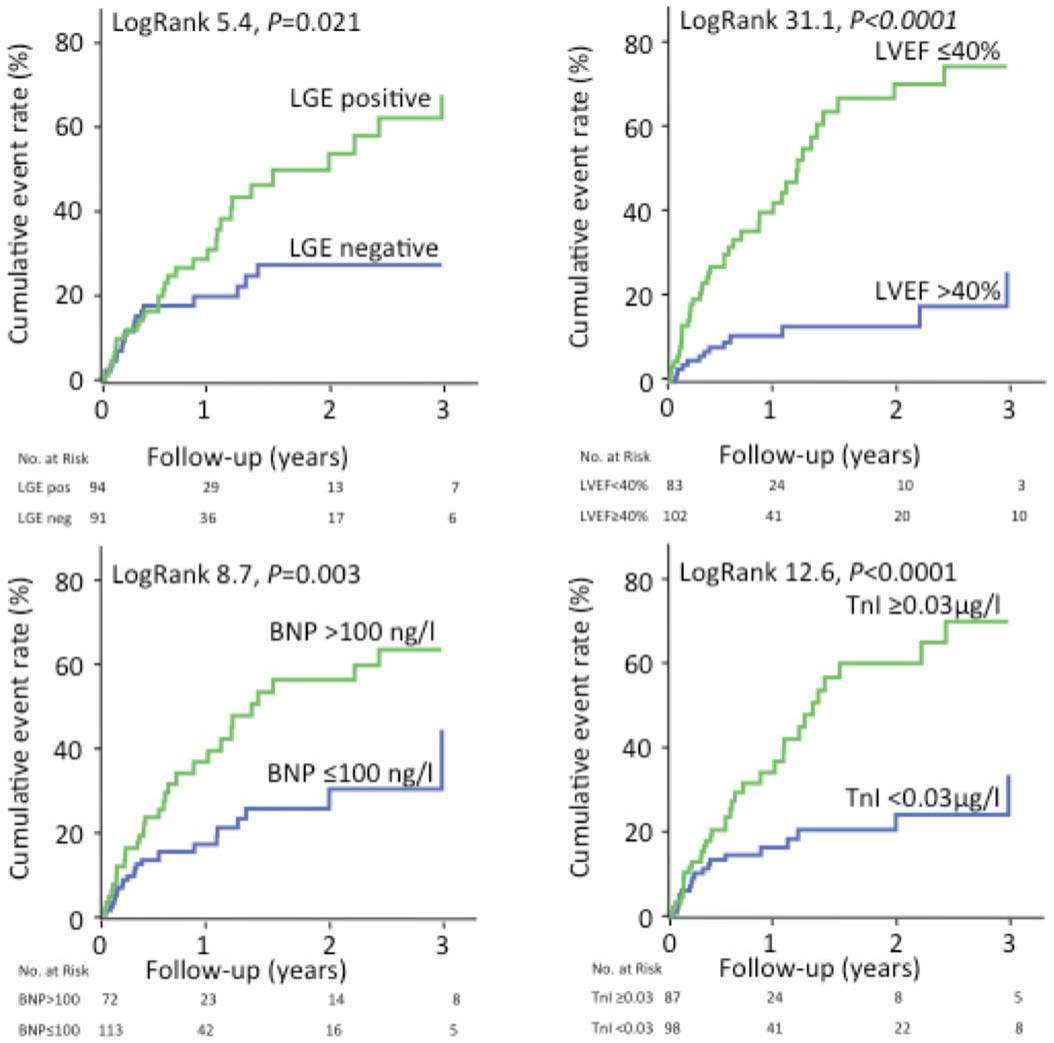
Kaplan-Meier-curves for prediction of the composite endpoint stratified by presence of late gadolinium enhancement, left ventricular ejection fraction, serum levels of brain natriuretic peptide and troponin I.

On univariable analysis, presence of LGE in CMR was significantly associated with the composite endpoint (Hazard ratio (HR) of 1.9 (1.1–3.4); p = 0.023). Cumulative 1, 2 and 3-year event rates were 30.8%, 49.7% and 67.4% in LGE-positive and 19.7%, 27.2% and 27.2% in LGE-negative patients, respectively (p = 0.021). Univariably, further risk markers were significantly associated with the composite endpoint on, namely BNP>100 ng/l (HR 2.2; 95% CI 1.3–3.8; p = 0.004), TnI ≥0.03 µg/l (HR 2.7; 95% CI 1.5–4.7; p = 0.001), LVEF ≤40% (HR 5.0; 95% CI 2.7–9.4; p<0.0001), LVEDD >55 mm (HR 3.0; 95% CI 1.8–5.1; p<0.0001) and QRS duration >98 ms (HR 1.9; 95% CI 1.1–3.3; p = 0.017) (table 5, figure 2).

**Table 5:** Hazard ratios for prediction of composite endpoint*.

	Univariable Analysis		Multivariable Analysis#	
Variable	HR (95% CI)	*P*	HR (95% CI)	*P*
Presence of LGE	1.9 (1.1–3.4)	0.023	1.1 (0.6–2.1)	0.676
NYHA ≥II	1.4 (0.8–2.5)	0.272		
BNP>100 ng/l	2.2 (1.3–3.8)	0.004	0.9 (0.5–1.8)	0.770
TnI ≥0.03 µg/l	2.7 (1.5–4.7)	0.001	2.2 (1.2–4.0)	0.014
LVEF ≤40%	5.0 (2.7–9.4)	<0.0001	3.9 (1.9–8.1)	<0.0001
LVEDD >55 mm	3.0 (1.8–5.1)	<0.0001	1.2 (0.6–2.4)	0.525
QRS >98 ms	1.9 (1.1–3.3)	0.017	1.2 (0.7–2.2)	0.473

* all-cause death, heart transplantation, aborted sudden death, sustained ventricular tachycardia, hospitalization due to decompensated heart failure.

# adjusted for age and gender.

BNP – brain natriuretic peptide, LGE – late gadolinium enhancement, LVEDD – left ventricular enddiastolic diameter, LVEF - left ventricular ejection fraction, TnI –troponin I.

On multivariable analysis, however, only LVEF ≤40% and TnI ≥0.03 µg/l were significant and independent predictors of outcome yielding relative risks of 3.9 (95% CI 1.9–8.1; p<0.0001) and 2.2 (95% CI 1.2–4.0; p = 0.014), respectively. Of note, presence of LGE was no independent risk predictor of outcome (HR 1.1; 95% CI 0.6–2.1; p = 0.676).

### Subgroup Analysis

When excluding patients with hypertrophic cardiomyopathy (n = 15) and patients with storage disease (n = 3), predictors of outcome of the remaining 102 patients with DCM and 65 patients myocarditis in univariable analysis were presence of LGE (HR 1.8; 95% CI 1.0–3.3; p = 0.040), BNP>100 ng/l (HR 2.0; 95% CI 1.2–3.6; p = 0.013), TnI ≥0.03 µg/l (HR 3.0; 95% CI 1.7–5.3; p<0.0001), LVEF ≤40% (HR 5.4; 95% CI 2.8–10.6; p<0.0001), LVEDD >55 mm (HR 3.0; CI 1.7–5.2; p<0.0001) and QRS duration >98 ms (HR 2.0; 95% CI 1.1–3.5; p = 0.017). However, in multivariable analysis, again, only LVEF ≤40% and TnI ≥0.03 µg/l were independent predictors of outcome yielding relative risks of 3.9 (95% CI 1.8–8.5; p = 0.001) and 2.4 (95% CI 1.3–4.5; p = 0.008), respectively.

## Discussion

The findings of the present study indicate that: i) In a cohort of consecutive patients presenting for evaluation of newly diagnosed non-ischemic cardiomyopathy prognosis of LGE-positive patients is significantly worse compared to LGE-negative patients; ii) multivariable analysis identifies only impaired LVEF (≤40%) and elevated TnI (≥0.03 µg/l), but not presence of LGE, as independent predictors of poor outcome; iii) Compared to LGE-negative patients, LGE-positive patients have lower LVEF, higher LVEDD and higher serum levels of BNP.

During the last decade, contrast-enhanced CMR emerged as important diagnostic tool for evaluation of myocardial diseases [15]. In healthy myocardium, gadolinium is washed out rapidly, whereas in damaged tissue it remains enriched due to its greater distribution volume [16]. Therefore, LGE imaging is considered to be non-invasive method for *in-vivo* assessment of myocardial infiltration, fibrosis and necrosis [17]. In recent years, various studies demonstrated not only a diagnostic but also prognostic role of contrast-enhanced CMR. In different entities of cardiac diseases including ischemic heart disease, DCM [10], [18], [19] and HCM [11], [20]–[22] presence of LGE was shown to be associated with susceptibility for malignant tachyarrhythmias as evidenced by increased ICD discharges or enhanced inducibility in electrophysiologic studies [23], adverse clinical course such as increased rate of rehospitalizations due to decompensated heart failure and also mortality [8], [24]–[27].

Previous studies focused on selected, clinically stable patients at a chronic stage of a predefined myocardial disease [10], [19]. However, these data do not reflect a real clinical world stetting where a large number of patients with newly diagnosed non-ischemic cardiomyopathy present, where the patient is acutely affected and where the underlying etiology of non-ischemic myocardial disease is not yet clear. Our study overcomes these shortcomings as we assessed the prognostic value of LGE in consecutive, unselected patients who presented for evaluation of acute heart failure symptoms of non-ischemic origin. In our cohort, LGE-positive patients had worse prognosis, but impaired left ventricular performance and elevated levels of cardiac biomarkers were stronger predictors of outcome. Of note, presence of LGE was not independently associated with outcome in multivariable analysis. This may be attributed to the fact that the value of LGE is associated with the underlying disease and might be an epiphenomenon of a damaged left ventricle.

Various other risk factors including left ventricular systolic dysfunction [28], increased left ventricular diameter, elevated levels of cardiac biomarkers [29] and QRS prolongation [30], [31] yielded prognostic information also in our cohort. On multivariable analysis, however, impairment of LVEF and elevated levels of TnI were the only independent predictors of adverse events while LGE, increased LVEDD and elevated levels of BNP did not independently contribute to risk prediction. Nevertheless, absence of left ventricular dysfunction did not assure freedom from adverse events. Seven patients with LVEF>40% reached an arrhythmic endpoint (5 discharges of ICD and 2 documentations of sustained ventricular tachycardia). All of these patients were LGE-positive, underscoring the concept that LGE may form a substrate for lethal ventricular arrhythmias [32].

Limitations of our study need to be recognized. The sample size of our study is limited. It cannot be ruled out that LGE would have been an independent predictor of outcome if a larger number of patients would have been included. Our study was designed to test the predictive value of LGE in “all-comers” with newly diagnosed non-ischemic cardiomyopathy. Therefore, our study cohort includes patients with different etiologies (DCM, myocarditis, hypertrophic cardiomyopathy and storage disease). This must be interpreted with care because it is well known that the underlying cause of non-ischemic cardiomyopathy itself also has strong prognostic importance [33] and because LGE should always be interpreted in the context of the specific etiology of the cardiomyopathy. Similarly, the conclusions of the current study that LVEF and cardiac biomarkers are the strongest independent predictors of adverse outcomes apply only to a heterogeneous cohort of unselected patients with newly diagnosed cardiomyopathy, and do not necessarily apply to individual patients or subgroups with newly diagnosed cardiomyopathy, where the prognostic implications of LGE, LVEF and cardiac biomarkers could vary significantly depending on the specific etiology of the cardiomyopathy. However, when patients with suspected hypertrophic cardiomyopathy and with storage disease were excluded, the primary finding of our study remained unaffected with LGE being a significant predictor of outcome only in univariable analysis and with impaired LVEF being the strongest independent risk predictor. In accordance with previous studies [11], [18], [19] we used a composite endpoint which also included non-life-threatening but clinically highly relevant events. To address true life-threatening endpoints a substantially larger cohort of patients with longer follow-up would be required. Forth, our study did not include several other risk predictors including such as markers of cardiac autonomic dysfunction [34]–[36] or t-wave alternans [37] which might be of prognostic importance in patients with newly diagnosed non-ischemic cardiomyopathy. Finally, we did not perform quantitative analysis of LGE.

### Conclusions

Data of the current study imply that in a hospital-based cohort of non-selected patients with newly diagnosed non-ischemic cardiomyopathy due to various etiologies presence of LGE on contrast-enhanced CMR is a risk predictor only in univariable analysis. However, LGE was not an independent predictor of outcome. Impairment of left ventricular function and elevated levels of troponin were the only independent risk predictors.
